# Human epidermal growth factor receptor 2 overexpression in gastric and gastroesophageal junction adenocarcinoma in patients seen at the University Teaching Hospital, Lusaka, Zambia

**DOI:** 10.4314/ahs.v20i4.41

**Published:** 2020-12

**Authors:** Chimwasu Kasochi, Peter Julius, Isaac Mweemba, Violet Kayamba

**Affiliations:** 1 University Teaching Hospital, Department of Pathology, Nationalist Road, Private Bag RW1X, Lusaka, Zambia; 2 The University of Zambia, School of Medicine, Nationalist Road, PO Box 50110, Lusaka, Zambia; 3 Tropical Gastroenterology & Nutrition Group, Department of Internal Medicine, PO Box 50398, Nationalist Road, Lusaka, Zambia

**Keywords:** Gastric and Gastroesophageal junction adenocarcinoma, Human Epidermal growth factor Receptor 2 overexpression, immunohistochemistry

## Abstract

**Background:**

There are scanty data on the occurrence of gastric tumours overexpressing human epidermal growth factor receptor 2 (HER2) in Africa.

**Objective:**

To assess HER2 protein overexpression in gastric and gastroesophageal junction adenocarcinoma (GGEAC) samples from a single centre in Zambia.

**Methodology:**

This was a cross-sectional study of formalin-fixed paraffin-embedded blocks with GGEAC. Prepared slides were first stained with Haematoxylin and Eosin and then evaluated for HER2 protein overexpression by immunohistochemistry.

**Results:**

A total of 57 gastric tissues were stained and evaluated for HER2 overexpression. Thirteen (23%) showed overexpression, 41/57 (72%) had negative and 3/57 (5%) had equivocal staining. The equivocal cases were excluded from the final analysis. Of the remaining 54 tissues, 28 (52%) were from females, and 26 (48%) were from males. The mean age was 59 years (SD 15 years). HER2 overexpression was highest in moderately differentiated tumours (p=0.0005). Intestinal type tumours had a higher occurrenc of HER2 overexpression than diffuse or mixed sub-types (p=0.0087). HER2 overexpression was not associated with age (p=0.27), sex (p=1.00) or anatomical location (p=1.00).

**Conclusion:**

The occurrence of GGEAC HER2 overexpression in Zambian patients is similar to proportions reported elsewhere, and it is associated with moderately differentiated tumours of the intestinal type.

## Introduction

Gastric cancer is one of the leading causes of cancer-related deaths worldwide. There were over one million gastric cancer cases in 2018, with an estimated 783 000 deaths. 1 The estimated age-standardised incidence rate in Africa is between 2.6 and 5.4 per 100 000. 2 In Zambia, gastric cancer is the tenth most common cancer, with an estimated age-standardised incidence of 2.9 per 100 000 and 3.4 per 100 000 in women and men respectively. 2

The most common type of gastric cancer is adenocarcinoma,^3^ which can either be located within the stomach or at the gastroesophageal junction referred to as gastric and gastroesophageal junction adenocarcinomas (GGEAC). GGEAC sub-types can be diffuse, intestinal or mixed Lauren classification). 4 Intestinal type adenocarcinoma is characterised by cohesive cells that form gland-like structures. Tumour cells that lack cell-to-cell interactions and infiltrate the stroma as single cells or small subgroups of non-cohesive, scattered tumour cells characterise diffuse adenocarcinomas. The mixed types have both the intestinal and diffuse characteristics.

A subset of advanced GGEAC overexpress HER2 protein, and these are aggressive, exhibit resistance to conventional chemotherapy and have poor outcomes. HER2 protein is a tyrosine kinase receptor, which is encoded by the HER2 proto-oncogene located on chromosome 17q21. HER2 is a member of the epidermal growth factor receptors (EGFRs) family, which is composed of four members, namely HER1, HER2, HER3 and HER4.^5^ Tumours with HER2 overexpression respond to targeted therapy with the anti-HER2 antibody trastuzumab and this treatment is associated with improved survival. 6 HER2 overexpression varies in different regions of the world with reported high expression in India (44%) 7 and low expression in China (6.9%). 8 In another study, Van Cutsem et al, reported similar proportions of HER2 overexpression from Europe (23.6%) and Asia (23.9%) but slightly lower from Central /South America (16.1%). 9 HER2 overexpression rates were higher in intestinal (31.8%) than diffuse (6.1%) type gastric cancer.

Data on the occurrence of gastric tumours with HER2 overexpression in Africa are scanty. A study done in Egypt on eighty-five tumour tissue samples from patients with gastric cancer revealed a HER2 positivity proportion of 27%. 10 It was expressed more in intestinal and mixed-type, adenocarcinoma and moderately differentiated tumours. 10 In a Kenyan descriptive cross-sectional study of sixty-six biopsy and resected specimens of histologically diagnosed gastric cancers, HER2 overexpression rate was found to be 42%. In this study, HER2 overexpression was higher in intestinal than diffuse-type cancers, and it was not associated with gender, age or anatomical site. 11

The prevalence of HER2 overexpression in GGE-AC in Zambia is not known. A retrospective audit of endoscopy records done in Zambia showed evidence of an increase in gastric carcinoma diagnosis, particularly in people below the age of sixty years. 12 Despite the poor GGEAC outcomes in Zambia, 13 patients with advanced disease are treated with chemotherapy and radiotherapy without the option of targeted immunotherapy. The lack of knowledge about the prevalence of HER2 overexpression in GGEAC hinders any prospects of targeted therapies that have the potential of improving patient outcomes. In this study, we therefore, assessed HER2 protein overexpression in patients with GGEAC. The University of Zambia Biomedical Research Ethics committee reference number 005-06-18 approved this study.

## Methods

This was a cross-sectional study to evaluate HER2 overexpression in archived formalin-fixed paraffin-embedded (FFPE) tissue samples in patients diagnosed with GGEAC in the Department of Pathology, University Teaching Hospital (UTH), Lusaka, Zambia. All FFPE tissue blocks with a histological diagnosis of GGEAC from January 2015 to June 2018 were evaluated. We excluded sample blocks with inadequate tissue and extensive crush/ mechanical distortion.

### Data collection plan and tools

Socio-demographic and clinical data were obtained from the data-intensive system and applications (a laboratory data collection system) and endoscopy reports. Clinical data (age, gender and tumour location) and pathological parameters (histological type and grade) were entered in standardised collection sheets. Retrieved FFPE tissue blocks were processed according to the standard operating procedure at the UTH pathology laboratory. Briefly, FFPE tissue blocks were sectioned, mounted on slides, stained with Hematoxylin and Eosin and read to confirm the initial diagnosis. Tissues with adenocarcinoma were classified histologically according to Lauren classification (intestinal, diffuse or mixed type). Tumours were graded as well differentiated if they exhibited more than 95% glands (Grade I). Moderately differentiated tumours had 50 to 95% glandular pattern (Grade II), while poorly differentiated ones have a predominantly solid pattern with less than 50% glands (Grade III). Immunohistochemistry for detection of HER2 followed strict adherence to the manufacturer's instructions using Dako rabbit polyclonal antibody Dako rbbit polyclonal antibody (RbAHuC-erbB2), Santa Clara, USA. Briefly, antigen retrieval was done using citrate buffer at pH 6.0 in a pressure cooker at high temperature for 30 minutes. Endogenous peroxidase blocking was done with 3% hydrogen peroxidase. The antibody was used at a dilution of 1:600.

### Analysis and interpretation for the presence of HER2 overexpression

HER2 scoring was done according to the Hoffmann scoring criteria 14 (Table 1). Those whose score was either 0 or 1+ were negative, the ones with 2+ were equivocal, and the ones with 3+ were positive or showed HER2 overexpression. We excluded specimens with equivocal results in the final analysis. Four independent pathologists (CK, CM, FM and PJ) did the analysis and interpretation of stained slides. For each of the slides, a score reported by three or more of the pathologists was recorded as the final score. In case of a discordant outcome (less than three similar scores), the score reported by PJ was taken as the tiebreaker as he was the most experienced pathologist. The kappa-statistic measure of interrater agreement between each of the pathologists was as shown in Table 2.

**Table 1:** Hoffmann scoring criteria for HER2 14

Score	Interpretation	Criteria for GGEAC
0	Negative	No staining or membrane staining in clusters of <5 tumour cells.
1+	Negative	Cluster(s) of at least five cohesive tumour cells with weak (generally visible early at X400 magnification) complete, basolateral or lateral membrane staining, irrespective of tumour volume percentage.
2+	Equivocal	Cluster(s) of at least five cohesive tumour cells with moderate (generally visible at X100- X200 magnification complete, basolateral membrane staining, irrespective of tumour volume percentage.
3+	Positive	Cluster(s) of at least five cohesive tumour cells with strong (generally visible at X25- X50 magnification) complete, basolateral or lateral membrane, staining irrespective of tumour volume percentage.

**Table 2:** Interrater agreement between pathologists' interpretation of immunohistochemically stained slides for HER2

Pathologist	CM	FM	PJ
CK	Kappa- 0.62 Agreement- 82%	Kappa- 0.54 Agreement- 71%	Kappa- 0.75 Agreement- 86%
CM	-	Kappa- 0.48 Agreement- 65%	Kappa- 0.50 Agreement- 73%
FM	-	-	Kappa- 0.43 Agreement- 62%

## Statistical analysis

Continuous variables were normally distributed and therefore summarised as mean, standard deviation and range. Categorical variables were summarised as frequencies and percentages. The Kappa statistics were employed to assess interrater agreement. Fisher's exact test was used to determine the significance of the association between HER2 overexpression and clinicopathological parameters. Results were then presented as odds ratios with 95% confidence intervals. A two-sided p-value of <0.05 was considered statistically significant. The data were analysed using STATA 15 (College Station, TX, USA).

## Results

The laboratory electronic database had records for 86 gastric and gastroesophageal junction adenocarcinomas (GGEAC) for the period under study (Figure 1). Seventy-three blocks were found in the archives and stained with Hematoxylin and Eosin. From these, 16 were excluded on account of inadequate tissue; a lack of tumour in the sections or extensive fragmentation rendering histologic interpretation impossible. Therefore, 57 samples were of good enough quality to allow for adequate immunohistological scoring. Of these, 55 cases were endoscopic biopsies, and 2 were gastric resections. Immunostaining for HER2 protein resulted in 13/57 (23%) positives (3+ or HER2 overexpression), 41/57 (72%) negatives (0 or 1+) and 3/57 (5%) equivocal (2+) results. Figure 2 shows representative images of HER2 expression in the variable grades of gastric and gastroesophageal adenocarcinoma.

**Figure:**
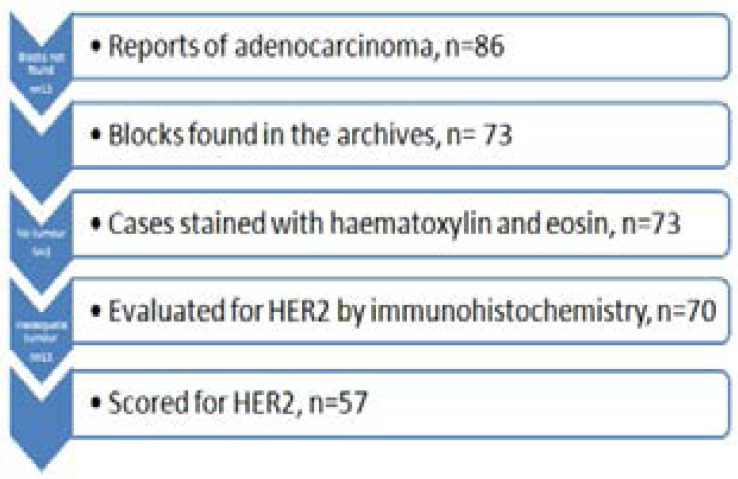


**Figure:**
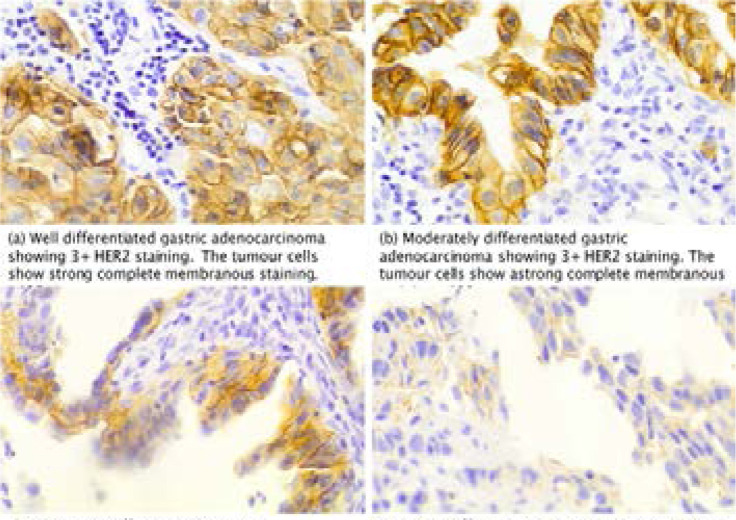


### Comparison of HER2 positive and negative patients

Demographic and histologic characteristics were compared between HER2 positive and negative tumours. For these analyses, equivocal specimens were excluded (n=3). Of the 54 specimens included, 28 (52%) were from females, and 26 (48%) were males. The mean age was 59 years (SD 15 years). Nine (17%) of the patients were below the age of 45 years. Of the 47 specimens whose anatomical location was knon, 4 (9%) were from the gastroesophageal junction, and the rest from gastric regions; 7 (15%) fundus, 20 (43%) body and 16 (34%) antrum. There was no association between HER2 overexpression and age (p=0.27), sex (p=1.00) or anatomical location of the tumour (p=1.00), (Table 3). Thirty-three 33 (61%) of the tumours were of intestinal, 16 (30%) diffuse and 5 (9%) mixed sub-types. Ninety-two percent of the tumours with HER2 over-expression were of the intestinal, 1 (8%) diffuse and none were of the mixed subtype. These differences were statistically significant, p=0.009 (Table 3). Poorly differentiated tumours were most common among those negative for HER2 staining, with only one being exhibiting overexpression. Similarly, this difference was statistically significant, p=0.0005, (Table 3).

**Table 3:** Patient and tumour characteristics for samples with either positive or negative HER2 staining

Patient or tumour characteristics	HER2 negative n=41 n (%)	HER2 Positive n=13 n (%)	95% Confidence Interval	*p*-value
**Sex** Male Female	20 (49) 21 (51)	6 (46) 7 (54)	0.2–4.4	1.00
**Age (years)** Below 30 30–44 45–59 Above 60	0 (0) 8 (20) 14 (34) 19 (46)	0 (0) 1 (8) 4 (30) 8 (62)	-	0.27
**Site (n=47)** Gastroesophageal junction Gastric (fundus, body and antrum)	3 (75) 31 (72)	1 (25) 12 (28)	0.02–12	1.00
**Histologic type** Intestinal Diffuse Indeterminate	21 (51) 15 (37) 5 (12)	12 (92) 1 (8) 0 (0)	-	0.009
**Tumour Differentiation** Well Moderately Poorly	2 (5) 14 (34) 25 (61)	3 (23) 9 (69) 1 (8)	-	0.0005

## Discussion

This study was undertaken to evaluate the prevalence of HER2 overexpression in patients with GGEAC presenting to the University Teaching Hospital in Lusaka, Zambia. Many GGEAC present with advanced disease and delayed referral is one of the major contributing factors. 15 In this study, there were slightly more females than males, but method of sampling does not allow for generalisation of the results to the Zambian population. Close to a quarter of the tumours examined in this study had HER2 overexpression. HER2 overexpression was variable depending on the histologic type and tumour differentiation. There was no association between HER2 overexpression and sex, age or anatomical location.

The proportion of HER2 positive GGEAC found in this study was less than that reported from Kenya, 11 another sub-Saharan African country. The Kenyan investigators had relatively more resection specimens while most of ours were endoscopic biopsies. Wand et al, reported a good concordance between biopsy and resection specimens for HER2 overexprssion in gastric cancer 16 therefore, specimen type is unlikely to be the reason for the observed difference. Our results were similar to those reported form Europe 17 and Egypt. 10 However, endoscopic biopsies do not allow for assessment of intra-tumoral HER2 heterogeneity. Intra-tumoral heterogeneity has been postulated as one of the factors that potentially influence therapeutic response of HER2 positive tumours. 18, 19

Anatomical site of the tumour was not associated with HER2 overexpression in this study, but the small sample size could have limited this. Other studies did, however, report similar findings.^8^, ^20^ Most of the tumours in this study were from the gastric region, predominantly distal portion. Available literature shows that distal gastric cancers are commoner in developing countries, among blacks of low socio-economic groups and are associated with Helicobacter pylori infection. 21

HER2 overexpression was reported to be positively associated with intestinal-type adenocarcinoma but inversely associated with E-cadherin mutations. 22 E-cadherin mutations are typical for diffuse gastric. 23 Here, we present similar findings. There is therefore, need to further investigate the selective overexpression of HER2 in intestinal-type gastric cancer.

Histological reporting of tumour differentiation is of limited clinical utility with no implications on treatment choices. Linking tumour differentiation grades to HER2 overexpression might be an avenue to improve its relevance. Information linking gastric tumour differentiation and HER2 overexpression have shown variable results with some studies showing an association,^24^,^25^, ^26^ while others have not. 20, 27

The anti-HER2 antibody trastuzumab, when added to chemotherapy, improves the survival of patients with HER2 positive GGEAC. 6 Trastuzumab is available in Zambia and patients with HER2 positive breast cancers receive this treatment. Gastric cancer patient outcomes in Zambia are very poor. 13 With the absence of data on the prevalence of HER2 overexpression in GGEAC currently, patients are not given the option of trastuzumab therapy. Results from this study have shown the need to test for HER2 overexpression in GGEAC in Zambia routinely. Incorporation of HER2 testing in the routine care of GGEAC patients and subsequent treatment with trastuzumab will contribute towards a better outcome for this sub-group of gastric cancer patients.

One limitation of this study was the small number of cases analysed due to the inability to retrieve all cases, and some blocks with inadequate tissue for analysis. Clinical data was incomplete for some cases. Being retrospective, we could not control for cold ischemic and fixation time. Lastly, we could not run fluorescent in-situ hybridisation on the three equivocal cases due to inadequate resources.

## Conclusion

HER2 overexpression in GGEAC is associated with intestinal-type and moderately differentiated tumours. There is a need to routinely test for HER2 overexpression in Zambian patients as its occurrence is similar to proportions reported elsewhere.
